# Molecular Mechanisms of Reelin in the Enteric Nervous System and the Microbiota–Gut–Brain Axis: Implications for Depression and Antidepressant Therapy

**DOI:** 10.3390/ijms25020814

**Published:** 2024-01-09

**Authors:** Ciara S. Halvorson, Carla Liria Sánchez-Lafuente, Jenessa N. Johnston, Lisa E. Kalynchuk, Hector J. Caruncho

**Affiliations:** 1 Division of Medical Sciences, University of Victoria, 3800 Finnerty Rd., Victoria, BC V8P 5C2, Canada; ciarahalvorson@uvic.ca (C.S.H.); carlaliria@uvic.ca (C.L.S.-L.); lkalynchuk@uvic.ca (L.E.K.); 2 Section on the Neurobiology and Treatment of Mood Disorders, National Institute of Mental Health, National Institutes of Health, Bethesda, MD 20892, USA; jenessa.johnston@nih.gov

**Keywords:** reelin, crypt–villus axis migration, enteric nervous system, microbiota, gut–brain axis, depression, antidepressant

## Abstract

Current pharmacological treatments for depression fail to produce adequate remission in a significant proportion of patients. Increasingly, other systems, such as the microbiome–gut–brain axis, are being looked at as putative novel avenues for depression treatment. Dysbiosis and dysregulation along this axis are highly comorbid with the severity of depression symptoms. The endogenous extracellular matrix protein reelin is present in all intestinal layers as well as in myenteric and submucosal ganglia, and its receptors are also present in the gut. Reelin secretion from subepithelial myofibroblasts regulates cellular migration along the crypt–villus axis in the small intestine and colon. Reelin brain expression is downregulated in mood and psychotic disorders, and reelin injections have fast antidepressant-like effects in animal models of depression. This review seeks to discuss the roles of reelin in the gastrointestinal system and propose a putative role for reelin actions in the microbiota–gut–brain axis in the pathogenesis and treatment of depression, primarily reflecting on alterations in gut epithelial cell renewal and in the clustering of serotonin transporters.

## 1. Introduction

Major depressive disorder (depression) is the leading cause of disability worldwide, with an estimated lifetime prevalence of 15% [1]). Current antidepressant treatments focus heavily on the monoaminergic system, relying on an imbalance in monoamines as the primary instigator of depression symptoms [2]. Therefore, a majority of antidepressant pharmaceuticals act to restore central monoamines to homeostatic levels. Approximately one third of patients, however, do not respond adequately to this therapy [3]. In recent years, there has been a shift towards exploring novel targets in the treatment of depression. Notably, the N-methyl-D-aspartate receptor (NMDAR) antagonist ketamine has fast antidepressant effects in treatment-resistant patients and offers an alternative to monoaminergic-centered antidepressants such as selective serotonin reuptake inhibitors (SSRIs) or serotonin and norepinephrine reuptake inhibitors (SNRIs) [4].

Another novel research stream is aimed at investigating enteric nervous system (ENS) dysregulation and interactions between the gut and the brain in depression. It is well known that gastrointestinal tract dysfunction occurs in depression, manifesting in symptoms such as abdominal pain, bloating, and diarrhea [5]. Some research suggests a causal role for the gut in the development of depression, highlighting leaky gut and dysbiosis as putative etiological factors [6,7]. As such, the gut is increasingly implicated in depression research and presents a novel perspective for antidepressant therapy.

The extracellular matrix (ECM) protein reelin is downregulated in central and peripheral structures in depression, and its levels are restored with the administration of antidepressants [8,9,10]. Restoring reelin to homeostatic levels has fast antidepressant effects parallel to those of ketamine in animal models of depression [11,12]. Moreover, reelin signalling appears to serve a pivotal role essential for the antidepressant effects induced by ketamine [13]. Altogether, these studies suggest that targeting the reelin system in some capacity might be a promising novel antidepressant treatment.

In the brain, reelin is an important regulator of cortical neuron migration during development, while in adulthood, it mediates synaptic plasticity, dendritogenesis, and synaptogenesis [14,15]. Outside of the brain, the main source of reelin is the liver, yet reelin expression has been found in several peripheral tissues including the intestines (here also referred to as “gut”) [16,17]. Reelin is present in the small intestine and colon [18] and plays a role in epithelial cell migration along the crypt–villus axis to facilitate proper turnover of the intestinal lining [16]. Moreover, some studies have suggested a role of reelin in the maintenance of the integrity of the intestinal barrier [16], as well as intestinal stem cell renewal and differentiation [19].

This review provides a comprehensive overview of reelin’s actions in the gut, delving into the potential molecular mechanisms through which reelin may influence the pathogenesis and treatment of depression, through its actions on the enteric nervous system and the microbiota–gut–brain-axis. While reelin seems to be involved in the pathogenesis of depression and might have antidepressant effects, research towards the involvement of reelin in the gut and in gastrointestinal dysfunction in depression is limited. The goal of this review is to guide future research towards exploring the interplay of reelin in the gut in the pathogenesis of depression.

## 2. Overview of Reelin

The name "reelin" originates from Falconer’s 1951 description of the "reeler" mouse phenotype observed in a laboratory in Edinburgh [20]. This phenotype emerged as a result of a spontaneous autosomal recessive mutation within a colony of inbred mice. These mice displayed an abnormal walking pattern characterized by a "reeling" gait, severe motor defects such as tremors and ataxia, and neuronal abnormalities in their brains. After years of studying these “reeler” mice, it was revealed that these alterations were caused by an autosomal recessive mutation in the reelin gene (*RELN*), leading to the absence of the reelin protein [20,21]. Consequently, these mice became known as reeler mice.

The heterozygous form of the reeler mouse (HRM) has since been extensively investigated to gain insights into neural developmental dysregulations and has proven invaluable in understanding the role of reelin in various psychiatric disorders. Studies with the HRM revealed that these animals displayed neural ectopia in the laminated structures of the brain, and from this observation, it was hypothesized that reelin might contribute to brain lamination. During development, reelin is secreted by Cajal–Retzius neurons in the marginal zone of the cerebral cortex where neuronal migration terminates and diffuses towards inner cortical layers [22,23]. Here, it regulates neuron organization and positioning—the initial processes of brain lamination [21,24,25]. 

Brain lamination is achieved through the migration of newborn neurons from neurogenic sites to the outer layers of the cortex. Radial glial processes extend through the cortex, providing a scaffold for inside-to-outside neuronal migration to form distinct cortical positionings; hence, this process is called radial migration [26]. Newborn neurons climb these radial glial processes until they are exposed to a stop signal and settle in their position. Early-born neurons settle in deeper cortical layers; later-born neurons navigate past them to occupy more superficial positions. Reelin is thought to serve as a chemoattractant for migrating neurons as it is diffusely present at the destination sites for migrating neurons [27]. In addition to acting as an attractant for migrating neurons, reelin equally serves as a signal for the termination of neuronal migration and detachment from radial glia [28,29]. As reelin is only present exteriorly to detached and settled neurons, it encourages proper cortical inside-out lamination. During the developmental process, reelin also plays a role in regulating cell aggregation and the formation of dendrites [30,31,32]. 

While essential in the developing brain, reelin is of comparable importance in the adult brain where it is predominantly secreted by γ-aminobutyric acid (GABA)ergic interneurons in the hippocampus and cortex, as well as by glutamatergic neurons in the cerebellum [33]. In this context, reelin assumes a new role as a neuromodulator, promoting synaptic plasticity, dendritogenesis, and the maintenance of neuronal structure [34,35,36]. Additionally, it plays a vital role in enhancing long-term potentiation processes that contribute to cognitive ability and the modulation of synaptic function [35,37,38]. 

Most of reelin’s functions during development and adulthood depend on the core reelin signalling pathway, which includes reelin canonical receptors very-low-density-lipoprotein receptor (VLDLR) and apolipoprotein E receptor 2 (ApoER2). Through binding to VLDLR and ApoER2, tyrosine residues on the intracellular adaptor protein disabled-1 (Dab1) are phosphorylated and initiate a downstream signalling cascade [39,40]. Dab1 performs various functions in the regulation of the cytoskeleton, cell migration, NMDAR modulation, synaptic plasticity, and dendritic spine growth [14,34,41]. During development, once neuronal migration is complete, neurons undergo a phase of more intricate dendritic branching and, in the process, they differentiate and mature, forming dendritic spines to establish synaptic connections with neurons in various circuits [42,43]. This maturation process involves dendritic pruning and modifications in the protein makeup of synapses, influenced not only by reelin’s canonical pathways but also by non-canonical pathways. Reelin binds to non-canonical receptors ephrin receptor ephrin type-B receptor 2 (EphB2) and α3β1-integrins. Through binding to EphB2, it controls the positioning of Cajal–Retzius cells during brain development [44] via cytoskeleton remodelling [45,46] and mediates NMDAR subunit composition and synapse function [14,47,48,49,50]. Via the canonical Dab1/CrkL/C3G/Rap1 pathway, reelin also influences the cytoskeleton [51]. Silencing or knocking down Dab1 rescues defective migratory processes, further substantiating that Dab1 is a positive regulator of cell migration [24].

Reelin expression extends beyond the central nervous system (CNS), encompassing various regions in the body throughout development and into adulthood, exerting influence through both canonical and non-canonical pathways. During development, reelin was primarily observed in the yolk sac and blood vessels, and in adulthood, it persists in the kidney, liver, testis, ovary, and adrenal and pituitary glands, as well as in blood cells [17,52,53]. This comprehensive view highlights reelin as a versatile molecule with multifaceted functions in both the brain and peripheral tissues. A detailed revision of reelin’s functions in the ENS is presented in the following section of this review.

## 3. Reelin in the Enteric Nervous System and Microbiota–Gut–Brain Axis

The enteric nervous system (ENS) is a complex network of neurons located within the walls of the gastrointestinal tract, structurally organized into two systems of interconnected parasympathetic ganglia: the myenteric (Auerbach’s) plexus and the submucosal (Meissner’s) plexus. The myenteric plexus regulates gut motility and coordinates peristaltic movements while the submucosal plexus plays a crucial role in hormone secretion, blood flow, and nutrient absorption. The ENS operates independently of the CNS and parallels the CNS in terms of functionality, neurotransmitter production, and morphology.

**Figure 1:**
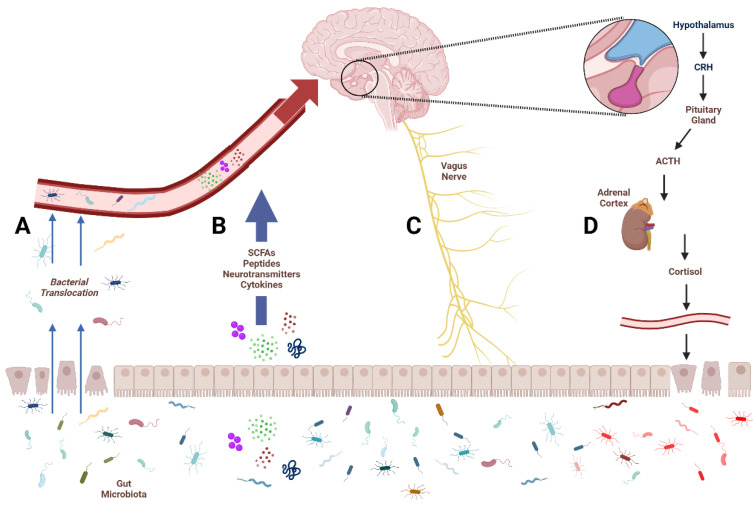
Avenues of communication between the gut and the brain. The four primary avenues of microbiota–gut–brain communication. Damaged epithelium leads to increased translocation of gut bacteria into circulation (**A**). Microbial metabolites like short-chain fatty acids (SCFAs), neurotransmitters, and cytokines enter circulation to affect central structures (**B**). The vagus nerve provides a bidirectional communication pathway for the gut and brain (**C**). Activation of the hypothalamus–pituitary–adrenal (HPA) axis leads to the production of cortisol, which increases intestinal permeability and alters microbial composition (**D**).

The microbiota–gut–brain (MGB) axis describes a complex and dynamic communication system between the brain, the gastrointestinal tract, and the vast community of microbes that reside in our gut. The gut microbiota produces various neuroactive compounds that influence numerous host functions; conversely, the brain influences gut and microbiome activity through autonomic innervation, endocrine signalling, and immune system function (Figure 1) [54]. This bidirectional axis has significant implications in the regulation of stress, mood, cognition, and pain, as well as in the pathogenesis of various physical and psychological disorders. In more recent years, the MGB axis has emerged as a putative target for the treatment of neuropsychiatric conditions such as depression [55,56]. In the context of the MGB axis, the ENS facilitates bidirectional communication by interacting with both the CNS and the gastrointestinal tract.

Homeostasis of the intestinal lining relies on maintaining harmonious interplay between cell growth, specialization, and cell death. Intestinal myofibroblasts play a role in maintaining these functions by secreting a diverse array of ECM components, including various cytokines, hormones, and neurotransmitters, and present a hub for communication between the basement membrane and the overlying epithelium [57,58]. Reelin, its canonical receptors VLDLR and ApoER2, effector protein dab1, and non-canonical receptors α3β1-integrins and EphB2 are all present in the small intestine and colon. Reelin is expressed in all intestinal layers as well as in submucosal and myenteric ganglia [18,19]. Interestingly, reelin mRNA is only expressed in myofibroblasts underlying the intestinal epithelium [59], but reelin receptors (ApoER2 and VLDLR) are expressed in myofibroblasts, epithelial cells [16], crypt cells, and enterocytes on the villi [18,19,59]. Specifically, ApoER2 was found exclusively in the apical portion of the villi, and VLDLR and Dab1 were observed predominantly in the apical zones of villi, as well as in the crypt [59] (see Table 1). 

Non-canonical reelin receptors EphB2 and α3β1-integrins are also expressed in the small intestine and colon [59,60,61,62]. Firstly, EphB2 is expressed in epithelial cells in the small intestine and colon tissue at elevated levels in the fetal period and continues to persist through adulthood [62]. α3 integrins are expressed in intestinal enterocytes and crypts and β1 integrins are expressed in intestinal myofibroblasts [19,59] (See Table 1). EphB2 is involved in cellular differentiation [62], while integrins play a role in connecting the ECM to the cytoskeleton. Specifically, α3β1-integrins can bind laminins, crucial basement membrane molecules in the ECM, to provide structural and biochemical support to cells [63] and also help in migration processes in the gut [64].

**Table 1:** Summary of the literature described in the text regarding the localization of reelin and its receptors in the gut.

Molecule	Organ	Cell Type	Model	Reference
Reelin protein	Small intestine	Myofibroblasts, crypt cells	Mouse, rat, human	[18,19,59]
	Colon	Ganglia, intramuscular nerve fibres	Mouse, rat, human	[16,18]
VLDLR receptor	Small intestine	Myofibroblasts, epithelial cells, crypt cells, enterocytes	Rat, human	[18,19,59]
	Colon	Myenteric ganglia	Mouse, rat, human	[18]
APOER2 receptor	Small intestine	Myofibroblasts, epithelial cells, enterocytes	Rat, human	[16,18,59]
	Colon	Myenteric ganglia	Mouse, rat, human	
α3β1-integrin receptor	Small intestine	Myofibroblasts, enterocytes, epithelial cells, crypt cells	Mouse	[19,59,61]
	Colon	Epithelial cells	Human (in vitro)	[61]
EphB2 receptor	Small intestine	Epithelial cells, crypt cells	Mouse, human	[60,61,62]
	Colon	Epithelial cells	Human	[60,61,62]

**Figure 2:**
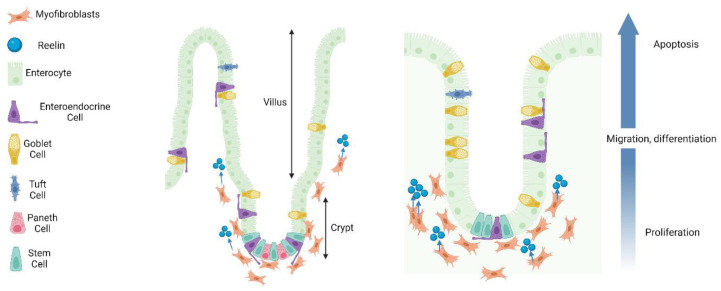
Crypt–villus axis migration in the small intestine and colon. A schematic delineating crypt–villus axis migration in the small intestine (**left** panel) and colon (**right** panel). Reelin is released from subepithelial myofibroblasts to aid in migration processes. Intestinal stem cells of the crypts differentiate into enterocytes, goblet cells, tuft cells, enteroendocrine cells, and Paneth cells (small intestine only). Migrating cells “push” adjacent cells above to encourage cell death and lining regeneration. Additionally, cell death on the villi facilitates proliferative and migratory processes in the crypts. Following migration to villus tips and differentiation, cells live for 3–5 days before being shed into the lumen of the gut. Paneth cells can live for close to 60 days.

Reelin’s role in CNS development is largely centered around the regulation of cell migratory processes; this likely comprises a large part of reelin’s function in the ENS as there are similar proliferation and differentiation processes along the crypt–villus axis. Although the mechanisms underlying crypt–villus cell migration are not comprehensively understood, they are believed to involve a combination of cell–cell adhesion, cell–matrix adhesion, and cytoskeletal changes. Several studies have demonstrated that reelin may also modulate cellular migration along the crypt–villus axis [16,59]. Parallel to its role in the CNS, reelin may function as an attraction protein for migrating neurons. One conflicting point, however, is that there is significantly more reelin expression in crypt lacteals as opposed to villi lacteals [19]. This can likely be attributed to the potential release of reelin at the basement membrane, with subsequent diffusion to the epithelium to carry out its functions. Taken together, these studies present a compelling case for the involvement of reelin in crypt–villus migration (Figure 2).

Homeostasis of the gut is maintained through reciprocal interactions between epithelial cells and the components of the ECM. Not surprisingly, a lack of reelin in reeler mice disrupts gut homeostasis. This disruption is characterized by a decreased number of Paneth cells, increased spaces between desmosomes and adherens junctions, and a change in the gene expression profile of the intestine [65,66]. While the precise mechanisms are not fully understood, it is plausible that reelin plays a role in regulating epithelial homeostasis by transmitting distinct signals through VLDLR or ApoER2 receptors following its secretion by intestinal myofibroblasts [59]. Furthermore, reeler mice experience more severe consequences following colonic injury [16], suggesting a protective function of reelin in preserving intestinal barrier integrity. 

Lastly, reelin likely plays a role in intestinal stem cell renewal and differentiation occurring in the enteric system and this is regulated by the intestinal lymphatic system [67,68]. This is believed to be due to the presence of reelin in crypt lymphatic vessels and reelin receptors in intestinal stem cells [19]. Moreover, goblet cells are specialized epithelial cells that secrete mucins to maintain the intestinal mucus layer. Significantly lower populations of goblet cells are detected in homozygous reeler mice compared to wild-type mice [16], suggesting the involvement of reelin in the process of cell differentiation, partially mirroring its function in the CNS. Nevertheless, further research is necessary to fully elucidate reelin’s functions in the gut and ENS.

## 4. Microbiota–Gut–Brain Axis Dysfunction in Depression

Depression, or major depressive disorder, is a serious chronic neuropsychiatric disorder predominantly characterized by an ongoing depressed mood, anhedonia, fatigue, and alterations in cognition, sleep, and appetite [69]. As one of the leading causes of disability worldwide, depression poses significant clinical challenges as it differs broadly across patients in both its presentation and response to treatment [70]. Therefore, depression may be viewed more so as a heterogeneous collection of symptoms with various physical and mental bases than a unified, well-defined disorder. As such, numerous hypotheses exist to explain the underlying mechanisms of depression.

Two primary mechanisms through which gastrointestinal symptoms arise in depression are HPA axis hyperactivity and dysregulated vagus nerve signalling. In depression, the HPA axis is often more active than in healthy individuals. This leads to elevated cortisol levels. Cortisol increases intestinal barrier permeability and alters microbial composition; the exact mechanisms are not wholly elucidated. Intestinal epithelial cells do, however, express the glucocorticoid receptor, indicating that cortisol directly influences the gut epithelium to regulate permeability [71]. Some research also suggests the involvement of mast cells. When subjected to a stressor or exogenous corticotropin-releasing hormone, certain participants experienced heightened intestinal permeability [72]. Subsequent subgroup analysis showed that alterations in intestinal permeability occurred exclusively in participants with elevated cortisol levels. Interestingly, the administration of sodium cromoglycate, an inhibitor of mastocyte degranulation, prevented this enhanced permeability of the intestinal barrier. 

Composed of 20% efferent and 80% afferent fibres, the vagus nerve extends from the brainstem to signal bidirectionally between the gut and the brain [73,74]. The vagus nerve can be targeted in the treatment of depression via vagus nerve stimulation (VNS), which has shown antidepressant effects in treatment-resistant depression [75]. A year-long study using VNS in treatment-resistant patients with major depression showed a 46% response rate and a 29% remission rate [76]. In 2005, VNS was approved for the adjunctive long-term treatment of treatment-resistant adult patients with chronic or recurrent major depression. While certain studies have shown promising results, its efficacy remains unclear [77]. While the ENS functions largely independently of the CNS, the vagus nerve communicates quite directly with the ENS, largely through cholinergic signalling to influence peristalsis and other digestive functions [78]. Cholinergic-dependent vagus signalling pathways have been described to produce a reduction in intestinal inflammation and intestinal permeability through the reinforcement of tight junctions (reviewed in [79]). These regulations have an indirect influence on microbiota composition and function. Stress suppresses vagus nerve function [80], suggesting possible implications for gut disturbances in major depression. Vagal afferent neurons can be activated through ENS signalling and microbe-produced substances, such as serotonin, to provide the brain with information about gut motility, nutrient absorption, and gut microbiota composition, as well as the presence of specific molecules within the gut (reviewed in [81]). These signals can influence various aspects of brain function, including mood, emotions, and behaviour [82].

The recent literature has increasingly linked dysfunction of the microbiota and the ENS to the development and progression of a range of mood and psychotic disorders, with particular emphasis on those associated with stress [83,84]. There exist two prominent barriers to gut–brain communication: the intestinal wall and the blood–brain barrier (BBB). The microbiome plays a prominent role in maintaining the integrity of the gut barrier [85] and influences structural aspects of the BBB to regulate BBB permeability and tight junction protein expression [86]. Abnormalities in either of these barriers may contribute to gut–brain axis dysfunction and, in turn, the pathogenesis of numerous gastrointestinal and psychological conditions. 

Irritable bowel syndrome (IBS), marked by persistent abdominal pain and changes in bowel movements, often coexists with depression [87]. Bilateral olfactory bulbectomy in mice induces chronic depression-like and anxiety-like behaviours associated with microbial reorganization and altered colonic motility [88]. In animal models, stress alters the size and quality of mucous secretion [89], pro-inflammatory cytokine infiltration of the colon [90], and gut permeability [91]. These findings substantiate the link between stress-related disorders and gastrointestinal function, highlighting the need for further investigation into the ENS as a route of pathogenesis for depression. The next sections further explain the impacts of dysbiosis and barrier disruption in depression.

### 4.1. Alterations to the Gut Barrier and Blood–Brain Barrier Integrity in Depression

Two prominent barriers to communication between the microbiome and brain are the intestinal barrier and the BBB. The intestinal barrier comprises the intestinal wall, while the BBB refers to the neurovascular unit that inhibits the crossing of many molecules from circulation into the brain. If gut barrier integrity is disturbed, there may be increased translocation of harmful substances and bacteria from the lumen of the gut into the bloodstream, which can then directly interact with immune cells or enteric neurons [92].

Foreign substances in the body can trigger an immune response, resulting in heightened inflammation that can culminate to negatively affect brain function and gut–brain communication. This process is called “leaky gut” and may play a role in the pathogenesis of depression [7]. Consistent with this theory, a study found altered levels of the gut permeability markers zonulin and intestinal fatty acid binding protein in individuals who had recently attempted suicide. These changes were found to be correlated with both increased systemic inflammation and a greater severity of depression symptoms compared to depressive patients with no suicide attempt and healthy controls [93]. Furthermore, the administration of certain probiotics, particularly those of the genus Lactobacillus, reduced stress-induced gut leakiness [94,95].

We postulate that, in most cases, intestinal changes and leaky gut come secondarily to depression. Notably, hyperactivity of the HPA axis, which is commonly seen in depression, leads to increased cortisol release. Elevated cortisol can cause the gut lining to become more permeable, which leads to an increased passage of bacteria and foreign material into circulation to induce an immune response. Therefore, leaky gut exacerbates the severity of depression. Furthermore, the altered monoamine levels found in depression can contribute to dysfunction in gut motility and function. Specifically, serotonin is found at abnormal levels in the gut in depression; distinctly from central structures, multiple studies using animal models show significantly elevated serotonin levels in the gut in depression [96]. Gut serotonin levels normalize following fecal matter transplantation [97]. Models of functional gastrointestinal disorders likewise show elevated serotonin levels, which may be attributed in part to a reduction in epithelial SERT leading to reduced uptake to serotonin [98,99,100]. In the gut, serotonin produces its effects primarily through binding to 5-HT3 and 5-HT4 receptors to initiate gastrointestinal reflexes, secretory processes, and peristaltic contractions [101,102]. 

Conversely to research demonstrating that stress can intensify intestinal inflammation and permeability [103], other studies indicate that experimentally induced colitis can lead to the emergence of anxiety-like behaviours. A recently established animal model of leaky gut entails the administration of dextran sulfate sodium (DSS) at a lower concentration to previous models to prevent complete destruction of the intestinal barrier and to produce barrier permeability comparable to patient populations with leaky gut [104]. Building off this research, Sudeep and colleagues (2022) administered 5% DSS in mice drinking water to produce symptoms relevant to colitis and leaky gut [105]. In addition to producing intestinal barrier dysfunction, DSS administration resulted in anxiety-like behaviours in mice, as evidenced by observations in both the open field test and the elevated plus maze. In another commonly used animal model of colitis, the 2,4,6-trinitrobenzene sulfonic acid (TNBS) model, significantly increased intestinal permeability is similarly accompanied by anxiety-like behaviour [106]. These lines of research demonstrate that while it is likely that leaky gut comes secondarily to chronic stress or depression, there is a reciprocal relationship in which each exacerbates the severity of the other.

Further evidence for a mutual exacerbation of gut symptoms and depressive symptoms comes from a recent meta-analysis, in which approximately one quarter of patients with IBS have depressive symptoms or disorders [107]. One mechanism of gut–brain connection results from leaky-gut-dependent increased levels of systemic pro-inflammatory cytokines, which in turn activate the HPA axis through stimulating the release of corticotropin-releasing hormone from the hypothalamus [108]. A definitive causal link has not yet been elucidated for IBS originating from depression or vice versa, but the high comorbidity suggests significant interactions between manifestations of intestinal inflammation, gut barrier hyperpermeability, and depressive symptoms.

The BBB is also frequently dysregulated in depression, but it remains uncertain whether this disruption is a consequence of depression or if depressive symptoms arise in part as a result of barrier disruption [109,110,111]. It is hypothesized that stress and resulting inflammation play a role in the disruption of the BBB, potentially through cytokine regulatory action on tight junction protein expression [112,113]. Additionally, neuroimaging studies have elucidated BBB hyperpermeability in depression [114]. The number of tight junction proteins in the BBB are lowered in depression; reduced tight junction proteins in the BBB increases BBB permeability and induces a depression-like phenotype [109]. Following the chronic unpredictable model of stress (CUMS), mice exhibited BBB breakdown associated with stress vulnerability and this was accompanied by a loss of Claudin-5, the tight junction protein that makes up the majority of the BBB [115,116]. Both the intestine wall and BBB play active roles in gut–brain communication and may therefore provide potential areas of interest for further research into novel therapies for depression. 

### 4.2. Alterations to the Microbiota in Depression 

Individuals with depression often present with alterations in the gut microbiome. Specifically, significant alterations in bacterial signatures of depressed patients were found upon analysis of bacterial composition compared to healthy controls [117]. Differences were observed in four bacterial phyla (Bacteroidetes, Proteobacteria, Firmicutes, and Actinobacteria) and sixteen bacterial families. A systematic review found significant microbial differences in the phyla Bacteroidetes, Firmicutes, Actinobacteria, Fusobacteria, and Protobacteria between depressive patients and healthy controls, with the phylum Firmicutes exhibiting the largest number of significantly different taxa [118]. Other systematic reviews described elevated levels of Actinobacteria in people with depression [119,120]. Conversely, Bifidobacterium and Lactobacillus were found to be significantly reduced in the microbiota of depressed patients compared to those of healthy controls [118,121,122], suggesting that the microbial dysbiosis associated with depression may not be entirely arbitrary. Notably, Bifidobacterium and Lactobacillus are among the most common microorganisms used in probiotics and have been shown to be beneficial for the treatment of depression [123].

Alterations in microbial metabolic function are also present in depression. Importantly, gut microbiota influences the production of serotonin via tryptophan metabolic pathways to affect numerous host functions, including behavioural and cognitive aspects associated with depression [124,125,126,127]. Short-chain fatty acids (SCFAs) modulate gut hormone secretion and gut motility, mitigate inflammation, and regulate microbial composition, homeostasis, and the circadian clock [54,128,129,130,131]. Additionally, SCFAs play an important role in gut–brain signalling and have been found to be depleted in depression [132,133,134,135]. The ratios of the three primary SCFAs in the colon differed in depressive individuals compared to controls without a psychiatric diagnosis [136]. The administration of SCFAs in mice prior to exposure to repeated psychosocial stress counteracted depressive-like behaviour and blunted the stress-induced corticosterone (CORT) spike compared to the non-stressed mice [137]. Additionally, microbial groups such as the Lachnospiraceae family, which participate in the breakdown of carbohydrates into SCFAs, have been found to be decreased in depression [138,139]. It has been elucidated that a decrease in fermentation-related microbes and a subsequent reduction in SCFAs contribute to disturbances in intestinal barrier integrity [140]. Therefore, SCFA alterations found in depression likely contribute to gastrointestinal and metabolic issues associated with depression [141].

Chronic stress has been widely recognized as a significant factor in the etiology of depression. Consequently, researchers often elicit chronic stress to induce depressive-like phenotypes in animal models. In more recent years, the link between chronic stress and the microbiome has become increasingly evident, with exposure to stressors eliciting significant changes in microbiome structure and stability [142,143]. Furthermore, acute stressors can also impact microbial integrity; distinct alterations in colonic mucosa-associated microbial composition were found in mice following exposure to a single 2-hour social stressor [144]. Notably, the abundance of Lactobacillus was significantly diminished, aligning with the aforementioned findings of reduced Lactobacillus in individuals with depression [121]. Multiple animal studies using a chronic stress model of depression have found altered microbial signatures in depression-like conditions [142,145,146]. Preclinical findings are reflected in clinical analyses of the microbiome in people with depression [147,148].

### 4.3. Effects of Antidepressants on the Microbiota–Gut–Brain Axis

The impact of conventional (monoamine-based) antidepressants on the gut has been largely overlooked.

It has been observed that SSRIs affect serotonin signalling in the gut and possess antibacterial properties which contribute to dysfunction in the gastrointestinal tract [149,150]. Serotonin is an important regulator of gut motility [101]; it is plausible that disruption in the intestinal serotonin system is foundational to some adverse effects of SSRIs such as nausea, constipation, or diarrhea [150,151]. Gastrointestinal disturbances are highly prevalent in individuals with depression, and more severe digestive issues coincide with more severe cases of depression [5,152]. It is likely that the disruption of serotonin signalling in the gut plays a major role in the occurrence of gastrointestinal disturbances in depression as serotonin is crucial for proper gut motility, secretion, and general digestive function [153]. 

In one study, an absence of serotonin transporter (SERT) produced diarrhea and constipation in mice [154]. Likewise, SSRI administration induced slower processing in upper gastrointestinal regions and decreased stool production in mice [155]. Following 29 days of fluoxetine administration, mice showed significantly altered microbiomes, particularly in bacterial phyla implicated in the management of body weight such as the phylum Bacillota (synonym Firmicutes) [156]. Furthermore, a study investigating the effects of five different antidepressants of the classes SSRI, SNRI, or tricyclic observed significant reductions in both microbiome richness and diversity following chronic treatment with any one of the medications [150]. Antidepressant-induced dysbiosis may contribute to a plethora of side effects commonly observed in antidepressant treatment as gut bacteria directly influence digestion, nutrient extraction, and metabolism [157]. For example, weight management is often dysregulated during antidepressant administration, and many of the microbial communities found to be disrupted by antidepressants are involved in the regulation of body mass [158].

It is important to note, however, that there is evidence supporting a restorative role of SSRIs in the microbiome through an increase in serotonin in the gut. A recent meta-analysis examined six studies using SSRIs for the treatment of IBS, with eleven using TCAs, and one using both (totaling 1127 patients) [159]. The administration of antidepressants significantly ameliorated IBS symptoms, suggesting that antidepressants have substantial action in the gut. Sun et al., 2019, demonstrated restored microbial health with the administration of fluoxetine following exposure to chronic unpredicted mild stress in mice [160]. Therefore, the effects of SSRIs on the microbiome are likely multifaceted and may depend on microbe type, individual differences, and environment. Either way, it is evident that antidepressants can modulate the microbiome to produce various side effects such as gastrointestinal dysfunction and altered metabolism, and that this can play into individualized responses to antidepressant therapy.

## 5. Reelin in Depression and Putative Antidepressant Roles

The discovery of a substantial reduction in reelin expression in mood and psychotic disorders, combined with the knowledge that reelin plays a role in controlling crucial elements of adult brain plasticity, has spurred our research group and others to investigate the potential involvement of reelin in the development of depression [9]. The next sections summarize the research conducted over the years by our group and others, showing the different involvements of reelin in depression and its putative antidepressant properties. 

### 5.1. Reelin in Depression

Being the most widely recognized, the monoaminergic hypothesis explains depression in terms of a deficit in monoamine neurotransmission resulting in a depletion of serotonin, norepinephrine, and dopamine in the CNS [161]. The vast majority of current antidepressant pharmaceuticals have been developed according to this hypothesis, with the most commonly prescribed being SSRIs [2]. Current treatment for depression emphasizes the use of antidepressant pharmaceuticals along with supplementary psychotherapy; however, approximately one third of patients do not show a sufficient positive response to this treatment [3]. Additionally, as these antidepressant drugs are delayed in producing a clinical response, tangible functional recovery can take months to achieve [162]. Therefore, it is imperative that alternative treatments are explored with significant consideration given to the heterogeneous etiology of depression.

Reelin’s relevance to depression lies in its downregulation in neuropsychiatric disorders and its observed fast antidepressant-like action in preclinical animal models of depression [9,163]. Decreased levels of peripheral and central reelin have been observed in schizophrenia, bipolar disorder, and depression [163,164,165,166]. Furthermore, an alteration in reelin levels, reelin receptor VLDLR, and Dab-1 has been observed with the administration of psychotropic drugs in healthy rodents [167]. The antidepressant fluoxetine causes an increase in VLDLR and Dab-1 while antipsychotics like clozapine and the mood-stabilizing drug lithium cause a decrease in reelin and Dab-1 [167]. 

Another theory presents alterations in neuroplasticity as a basis for the development and progression of depressive symptoms. Functional plasticity changes to brain networks and structural plasticity changes, such as altered dendritic spine morphology, are observed in depression [168]. Specific brain regions, including the amygdala and basal ganglia, are often hyperactive in depressed patients, and depressed patients show different patterns of brain activation in the resting state [169,170]. During cognitive and emotional tasks, depressed patients show divergence in region activity compared to healthy controls [170]. Reelin is important for maintaining neuron stability and connections and in regulating synaptic plasticity. Individuals with depression present with reduced reelin levels, which may contribute to disrupted structural aspects of plasticity, such as alterations to dendritic spine morphology and synaptic stability [164]. These processes may culminate in abnormal (hyper- or hypo-)activation of brain regions and networks when compared with control participants. In addition to its effects in stabilizing neuron structure and regulating synaptic plasticity, reelin also orchestrates gut lining regeneration through controlling cell migration along the crypt–villus axis [65]. Reduced reelin in the gut leads to slowed intestinal cell migration and hindered lining renewal, which may contribute to the digestive issues that commonly accompany depression.

Our laboratory primarily uses a chronic stress model of depression, wherein repeated CORT injections are delivered to induce a depressive-like phenotype in rats [9,171]. CORT injections significantly increase immobility in the forced swim test (FST) and decrease sucrose consumption in the sucrose preference test; these parameters are rescued with an acute peripheral reelin injection [12]. Heterozygous reeler mice display increased susceptibility to the depressogenic effects of CORT compared to wild-type mice, suggesting that impaired reelin expression may increase vulnerability to glucocorticoids as a risk factor for the development of depressive-like symptoms [172]. In contrast, mice with an overexpression of reelin exhibit increased resilience following exposure to chronic stress compared to heterozygous reelin knockout mice [173]. Certain antidepressants have been shown to prevent or rescue CORT-induced decreases in reelin-positive cell count in the subgranular zone (SGZ) of the hippocampus, providing further validation for this paradigm [11,174].

The prevalence of treatment-resistant depression underscores the necessity for alternative approaches to the treatment of depression that offer faster and more reliable outcomes [3]. In recent years, subanaesthetic ketamine administration has emerged as a promising fast-acting antidepressant treatment. Its antidepressant mechanism is thought to involve the upregulation of α-amino-3-hydroxy-5-methyl-4-isoxazolepropionic acid receptor (AMPAR) transmission, leading to increased activation of the mechanistic target of rapamycin complex 1 (mTORC1) through downstream signalling pathways [175]. However, ketamine is associated with psychomimetic and dissociative symptoms and carries the risk of potential abuse [176,177,178]. There is therefore a growing interest in researching alternative therapeutics that operate through similar mechanisms but without the unwanted side effects believed to be linked to ketamine’s antagonistic effect on NMDAR. Reelin likewise activates mTORC1, but avoids NMDAR antagonism, suggesting that rescuing reelin to homeostatic levels may offer rapid antidepressant effects similar to those of ketamine while avoiding the potential for dissociative effects [12,179].

### 5.2. Putative Antidepressant Role of Reelin in the Enteric Nervous System

Although the administration of reelin has been shown to produce rapid antidepressant effects in the CNS, there is currently a lack of research demonstrating the effects of reelin administration in the ENS in the context of depression. As many of reelin’s effects in the CNS are mirrored in the ENS, and reelin and its receptors are present in the ENS, it can be postulated that reelin may have similar rapid antidepressant action in the ENS.

The link between the ENS and depression may be further substantiated by SERT clustering. A subset of intestinal epithelial cells called enterochromaffin cells produce serotonin and release it into the lumen of the gut. Serotonin is transported back into intestinal epithelial cells via SERT to regulate serotonin signalling within the gut [153]. As previously mentioned, the ENS contains close to 95% of the body’s serotonin stores, a neurotransmitter heavily implicated in the pathophysiology of depression [180]. The clustering of SERT into specific membrane domains is critical for serotonin reuptake activity [181], and lower reelin expression in homozygous and heterozygous reeler mice leads to an increase in the number and size of membrane protein clusters, including SERT, in peripheral lymphocytes [182]. Meanwhile, the introduction of recombinant reelin to synaptosomes modifies membrane protein clustering via binding to integrin receptors [183]. Moreover, in a chronic stress animal model of depression where reelin is decreased compared to controls, we observed increases in SERT cluster size that were normalized with reelin treatment both in vivo and in vitro [8,11]. Additionally, depressed patients present with differences in SERT clustering in peripheral lymphocytes compared to controls [182]. SERT clustering may therefore provide a biomarker of therapeutic response in treatment-naïve patients with depression. A lack of SERT is also linked to dysbiosis and alterations in the metabolic function of the mouse microbiome [184]. Furthermore, mice that underwent CUMS treatment had significantly lower levels of intestinal SERT [185]. It has not been ascertained whether deficits in gut SERT are restored with reelin; however, these findings suggest a putative role for reelin in altering SERT activity in the ENS.

Although there has been no direct investigation into intestinal reelin levels in depression, decreases in peripheral reelin have been observed in mood and psychotic disorders [164]. Therefore, it is plausible that reelin is likewise downregulated in the gut. In depression, there is a decreased intestinal stem cell proliferation rate and decreased cell migration along the crypt–villus axis, as evidenced by lower villus and higher crypt DNA levels in rats exposed to a chronic stress paradigm compared to healthy controls [186]. In line with this, epithelial cell proliferation in the small intestine was impeded in rats following prolonged cold restraint stress [187]. As reelin is important in the regulation of intestinal stem cell differentiation and crypt–villus migration, lower levels of intestinal reelin in depression likely contribute to the impairment of crypt–villus differentiation and migration processes observed in depressive subjects. In turn, this may translate into some of the gastrointestinal disturbances commonly experienced by individuals with depression. It would be interesting to investigate whether peripheral reelin administration could alleviate the severity of this dysregulation.

In the adult CNS, reelin promotes synaptic plasticity, dendritogenesis, and the maintenance of neuronal structure [35,36,188]. As many of reelin’s central functions are mirrored in the ENS, it is probable that reelin also plays a role in enteric neuron communication and architecture. Depression-induced reduction in gut reelin content may therefore produce weakened neuronal signalling to affect overall gut function. Impaired reelin action in the gut likely plays into the discombobulated digestive symptoms that often occur concomitantly with depression. Dysfunctional or weakened enteric neuron communication may additionally affect microbiome health. It has been demonstrated that an absence of enteric innervation results in microbial overgrowth and an overexpression of pro-inflammatory bacteria such as *E. coli* [189]. Moreover, disorders such as Hirschsprung disease, characterized by incomplete ENS development, are often accompanied by microbial abnormalities [190]. Therefore, the ENS likely plays a role in the maintenance of the microbiome. Interestingly, reeler mice exhibit increased hippocampal presynaptic vesicles accompanied by decreased SNAP25 expression, suggesting a role for reelin in presynaptic vesicle release [191]. As reelin is implicated in ENS function, reelin may also indirectly regulate microbiome composition and stability.

In light of these investigations, it is plausible that reelin is involved in the progression of many of the gut abnormalities observed in depression, and that there is a cyclic nature to these disruptions. In chronic stress and depression, there is increased inflammation and disruption in gut motility, gut lining, and microbial function, manifesting the gastrointestinal symptoms often associated with conditions of chronic stress. Stress likewise causes disruption in reelin expression, further contributing to the aforementioned gastrointestinal ailments, primarily through disruption to gut lining regeneration. Peripheral reelin injections have been shown to rescue various alterations in the brain following exposure to paradigms eliciting depressive-like symptomatology [8]. Additionally, both acute and repeated reelin injections restored chronic-stress-induced alterations in the spleen, a prominent immune organ [192]. As peripheral reelin injections have been observed to rescue both central and peripheral alterations in depressive-like models, it may be postulated that reelin injections may mitigate or reverse ENS and microbiota dysfunction in depression, providing a novel therapeutic avenue that operates through mechanisms distinct from most currently used antidepressants. However, further research should be conducted to more firmly elucidate reelin’s role in the gut in neuropsychiatric disorders and to explore the effects of peripheral reelin administration on intestinal tissues.

## 6. Conclusions

The treatment of depression has long been focused on balancing the dysregulation of monoamines in the CNS. The clinical management of depression presents significant challenges due to its wide variability in presentation and treatment response among patients. Reelin provides a novel avenue for research into therapy for depression as it has fast antidepressant effects that operate through mechanisms distinct from most currently used antidepressants. In the CNS, reelin regulates radial migration in cortical development, as well as synaptic plasticity and dendritogenesis in the adult brain. In recent years, the ENS and the gut–brain axis have been increasingly implicated in the pathogenesis of depression. Research has elucidated compositional and functional microbiome deficits and gut–brain axis dysfunction in depression. Reelin and its receptors (VLDLR, ApoER2, EphB2, and α3β1-integrins) are present in the gut. In the ENS, reelin regulates cellular migration along the crypt–villus axis via canonical and non-canonical signalling mechanisms and supports intestinal barrier homeostasis. Furthermore, intestinal stem cell proliferation and cell migration along the crypt–villus axis are dysregulated in depression. Although reelin research is currently restricted to preclinical testing, it is still necessary to assess and consider how reelin would best be administered to patients. As reelin has similar signalling to ketamine and parallels ketamine in treatment duration, its administration in the clinical setting would likely mirror the approach used for ketamine [12]. Currently, ketamine is used for treatment-resistant depression and is administered to patients either via a slow infusion over 40–60 min or using a nasal spray containing esketamine. Preliminary findings from our lab indicate observable effects of reelin one week post-injection [193], suggesting that infusions might be tolerable as they would not need to occur frequently. Based on the summarized literature, we conclude that there might be a role for intestinal reelin in the pathogenesis of depression and that reelin-based therapeutics could serve as a putative novel avenue better targeting the nature of depression at a multidimensional level. 

## Abbreviations

AMPA	α-amino-3-hydroxy-5-methyl-4-isoxazolepropionic acid
ApoER2	Apolipoprotein E receptor 2
BBB	Blood–brain barrier
CNS	Central nervous system
CORT	Corticosterone
CUMS	Chronic unpredictable model of stress
Dab1	Disabled-1
DNA	Deoxyribonucleic acid
DSS	Dextran sulfate sodium
ECM	Extracellular matrix
ENS	Enteric nervous system
EphB2	Ephrin type-B receptor 2
FST	Forced swim test
GABA	γ-aminobutyric acid
HRM	Heterozygous reeler mouse
HPA	Hypothalamus–pituitary–adrenal
IBS	Irritable bowel syndrome
MGB	Microbiota–gut–brain
mTORC1	Mechanistic target of rapamycin
NMDA	N-methyl-D-aspartate
SCFA	Short-chain fatty acid
SERT	Serotonin transporter
SGZ	Subgranular zone
SNRI	Serotonin and norepinephrine reuptake inhibitor
SSRI	Selective serotonin reuptake inhibitor
TNBS	2,4,6-trinitrobenzene sulfonic acid
VLDLR	Very-low-density-lipoprotein receptor
VNS	Vagus nerve stimulation
